# Comparative transcriptome analysis of three gonadal development stages reveals potential genes involved in gametogenesis of the fluted giant clam (*Tridacna squamosa*)

**DOI:** 10.1186/s12864-020-07276-5

**Published:** 2020-12-07

**Authors:** Jun Li, Yinyin Zhou, Zihua Zhou, Chuanxu Lin, Jinkuan Wei, Yanpin Qin, Zhiming Xiang, Haitao Ma, Yang Zhang, Yuehuan Zhang, Ziniu Yu

**Affiliations:** 1 Key Laboratory of Tropical Marine Bio-resources and Ecology, Guangdong Provincial Key Laboratory of Applied Marine Biology, South China Sea Institute of Oceanology, Chinese Academy of Science, 164 West Xingang Road, Guangzhou, 510301 China; 2 Southern Marine Science and Engineering Guangdong Laboratory (Guangzhou), Guangzhou, 510301 China; 3 Hainan Key Laboratory of Tropical Marine Biotechnology, Sanya Institute of Oceanology Chinese Academy of Sciences, Sanya, 572024 China; 4 Innovation Academy of South China Sea Ecology and Environmental Engineering, Chinese Academy of Sciences, Guangzhou, 510301 China; 5 University of Chinese Academy of Sciences, Beijing, 100049 China

**Keywords:** *Tridacna squamosa*, Gonadal development and gametogenesis, Transcriptome, Reproduction, Differential expression genes

## Abstract

**Background:**

Gonad development and differentiation is an essential function for all sexually reproducing species, and many aspects of these developmental processes are highly conserved among the metazoa. However, the mechanisms underlying gonad development and gametogenesis remain unclear in *Tridacna squamosa*, a large-size bivalve of great ecological value. They are protandrous simultaneous hermaphrodites, with the male gonad maturing first, eventually followed by the female gonads. In this study, nine gonad libraries representing resting, male and hermaphrodite stages in *T. squamosa* were performed to identify the molecular mechanisms.

**Results:**

Sixteen thousand four hundred ninety-one unigenes were annotated in the NCBI non-redundant protein database. Among the annotated unigenes, 5091 and 7328 unigenes were assigned to Gene Ontology categories and the Kyoto Encyclopedia of Genes and Genomes (KEGG) Pathway database, respectively. A total of 4763 differentially expressed genes (DEGs) were identified by comparing male to resting gonads, consisting of 3499 which were comparatively upregulated in males and 1264 which were downregulated in males. Six hundred-ninteen DEGs between male and hermaphroditic gonads were identified, with 518 DEGs more strongly expressed in hermaphrodites and 101 more strongly expressed in males. GO (Gene Ontology) and KEGG pathway analyses revealed that various biological functions and processes, including functions related to the endocrine system, oocyte meiosis, carbon metabolism, and the cell cycle, were involved in regulating gonadal development and gametogenesis in *T. squamosa*. Testis-specific serine/threonine kinases 1 (TSSK1), TSSK4, TSSK5, Doublesex- and mab-3-related transcription factor 1 (DMRT1), SOX, Sperm surface protein 17 (SP17) and other genes were involved in male gonadal development in Tridacna squamosal. Both spermatogenesis- (TSSK4, spermatogenesis-associated protein 17, spermatogenesis-associated protein 8, sperm motility kinase X, SP17) and oogenesis-related genes (zona pellucida protein, Forkhead Box L2, Vitellogenin, Vitellogenin receptor, 5-hydroxytryptamine, 5-hydroxytryptamine receptor) were simultaneously highly expressed in the hermaphroditic gonad to maintain the hermaphroditism of *T. squamosa*.

**Conclusion:**

All these results from our study will facilitate better understanding of the molecular mechanisms underlying giant clam gonad development and gametogenesis, which can provided a base on obtaining excellent gametes during the seed production process for giant clams.

**Supplementary Information:**

The online version contains supplementary material available at 10.1186/s12864-020-07276-5.

## Background

Reproductive development and sex determination are widespread and significant processes which have long been of interest to biologists. The processes of sex determination and differentiation are tremendously diverse in mollusks, ranging from functional (simultaneous) hermaphroditism, alternative sexuality (sequential hermaphroditism), strict gonochorism or dioecy (species that exist as separate males and females), to species that are capable of sex changes [1]. Giant clams (subfamily Tridacninae), the largest living bivalves in the world, are native to coral reefs throughout much of the tropical Indo-Pacific [2]. These organisms play various roles in coral reef ecosystems, for example, their shells act as substrates for epibionts, and serve as nurseries to various organisms [2]. All giant clams are protandrous functional hermaphrodites, becoming simultaneous hermaphrodites in later years. The male phase of the gonad develops first and eventually matures the female gonads. The normal spawning sequence is for sperm to be produced first, followed by egg production after a short interval. Release of sperm is triggered in nature by the presence of a spawning inducer associated with ripe eggs [3]. Unfortunately, giant clams have suffered from widespread harvesting for food, shell collecting and the aquarium trade. The over-exploitation of giant clams has led to the decline of the population throughout its geographic range and ecological extinction [4]. Thus, a certain degree of difference was found between the genetic structures of giant clam species [5, 6]. Consequently, all giant clam species are protected under the Convention of International Trade in Endangered Species of Wild Fauna and Flora (CITES) and are listed on the International Union for Conservation of Nature (IUCN) Red List of Threatened Species [7]. Therefore, better quality and higher seeds production are required to maintain the sustainable development of giant clams.

In order to control the quality and quantity of giant clams and their eggs in aquaculture, it is crucial to understand the molecular mechanisms of gonad development and gametogenesis, which may facilitate the production of high-quality clam seeds. The first step toward understanding molecular mechanisms of gonad development and gametogenesis is to identify and characterize reproduction-related genes and pathways. However, studies on gonad development and gametogenesis genes and pathways in mollusks are few and limited. In these previous studies, many efforts have been made to reveal genes homologous to sex-determining pathway genes in model species [8–10]. The vertebrate female-determining genes including β-catenin and forkhead box L2 (FOXL2), as well as male-determining genes including double-sex- and mab-3-related transcription factor (DMRT) and SOXE, have been identified in some mollusks. In *Crassostrea gigas*, CgFOXL2 expression increases during the adult gametogenetic cycle for both sexes, but with a significant increase occurring earlier in females than in males [11]. Cg-β-catenin is expressed in vitellogenic oocytes and may be involved in early oyster gonadic differentiation [12]. In *Chlamys nobilis*, CnDMRT2 is likely to be involved in playing a functional role in male gonadal development or maintenance of gonadal function, and CnDMRT5 may be involved in biological processes other than gonadal development in *C. nobilis* [13]. In *Pinctada martensii*, PmDMRT2 might play a functional role during spermatogenic cell differentiation from spermatocytes and spermatids into sperm [14]. However, unlike other families of bivalves, which have doubly uniparental inheritance (DUI) and sex reversal [15, 16], *T. squamosa* is a functional hermaphroditic bivalve [17]. In *T. squamosa*, sex is more likely to be dominated by the interaction of multiple genes. Next-generation sequencing technology has been utilized to study the genes related to reproduction in various species [18–24], but no data is currently available on the gonad transcriptome of *T. squamosa*.

In the present study, to obtain a comprehensive transcriptome database of the various gonad developmental stages in *T. squamosa*, we used the Illumina sequencing technology to discover genes potentially involved in gonad development and gametogenesis for resting, male, and hermaphroditic gonadal developmental stages. To our knowledge, this work is the first report on transcriptome profile analysis of gonads in *T. squamosa*. Results from the transcriptome analysis would be particularly important for improving understanding of the molecular mechanisms underlying the regulation of gonadal development and providing novel insights into the aquaculture of *T. squamosa*.

## Results

### Giant clam gonad development and histological observation

**Fig. 1:**
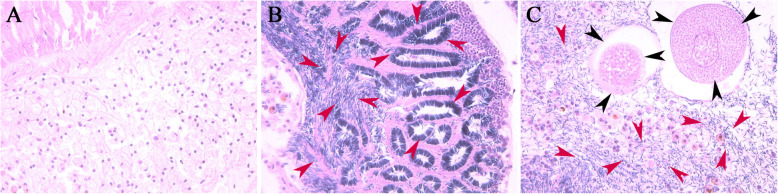
Developmental stages of *Tridacna squamosa* gonads by histology. Resting, male and hermaphrodite stages are presented in images **a**, **b**, and **c**, respectively. The red, and black arrows indicate sperm and oocyte. All histological section pictures were taken under multiple of× 40

To gain a better understanding of gonad development, histological analysis using HE-stained sections was conducted to compare different development stages. Histology showed that resting gonads are filled with connective tissue and lack any gamete-producing tissue or other tissue which could be associated with a particular sex. In the male gonads, the tissues were comprised of spermatogonia, primary spermatocytes, secondary spermatocytes, and spermatids. In the hermaphrodite gonads, both oocytes and sperm were detected (Fig. 1).

### Evaluation of biological replicates

Pearson’s Correlation Coefficient (r) is an important index for the evaluation of the correlation of the samples. Based on the r2 values in Table S2, two comparisons were made (resting versus male, male versus hermaphrodite) to avoid comparing significantly different samples, improving data authenticity and repeatability between samples.

### Sequencing and de novo assembly

In the present study, nine cDNA libraries were constructed for Illumina sequencing. The data processing results were summarized in Table 1. After eliminating primers, adapter sequences, and low-quality reads, a total of 43,251,171 clean reads were obtained from the resting gonads, 42,793,935 from the male gonads, and 38,375,061 from the hermaphroditic gonads. All clean data were assembled into 124,565 transcripts and 95,408 unigenes with a mean length of 872.13 and 746.29 bp, which exhibits a BUSCO transcriptome completeness of 78.4%. A total of 5089 (5.33%), 5091 (5.33%), 7328 (7.68%), 10,620 (11.13%), 13,622 (14.27%), 9289 (9.74%), 14,678 (15.38%), and 16,491 (17.28%) unigenes had significant matches with sequences in the COG, GO, KEGG, KOG, PFAM, Swissprot, eggNOG, and NR databases, respectively (Table 2). The annotation results showed that more than half (72.72%) of the genes were not well annotated, due to lacked significant similarity with other sequences deposited in the aforementioned databases.

**Table 1:** Summary statistics of *Tridacna squamosa* gonad transcriptome sequencing

Item	Raw reads	Clean reads	Mapping reads	Mapping efficiency (%)	Q30
Resting 1	22,158,743	14,964,323	8,961,631	59.89%	91.86%
Resting 2	21,613,669	14,772,577	8,963,849	60.68%	92.08%
Resting 3	21,913,936	13,514,271	8,092,445	59.88%	92.22%
Male 1	21,603,054	15,187,233	10,548,337	69.46%	92.49%
Male 2	21,036,966	14,859,379	9,963,548	67.05%	91.83%
Male 3	21,461,018	12,747,323	8,515,377	66.80%	92.06%
Hermaphrodite 1	21,101,077	12,955,705	7,897,579	60.96%	92.15%
Hermaphrodite 2	21,848,415	12,944,307	8,440,079	65.20%	92.06%
Hermaphrodite 3	21,446,006	12,475,049	8,885,081	71.22%	92.04%

**Table 2:** Statistics of assembly and annotation for *Tridacna squamosa*

Dataset name	Number
Assembly	
Number of transcripts	124,565
Mean length of transcripts (bp)	872.13
N50 length of transcripts (bp)	1488
Number of unigenes	95,408
Mean length of transcripts (bp)	746.29
N50 (bp) length of unigenes	1143
Annotation	
COG_Annotation	5089
GO_Annotation	5091
KEGG_Annotation	7328
KOG_Annotation	10,620
Pfam_Annotation	13,622
Swissprot_Annotation	9289
eggNOG_Annotation	14,678
nr_Annotation	16,491
All_Annotated	16,915
BUSCO Completeness	
Complete BUSCO	78.4%
Complete and single-copy BUSCO	45.2%
Complete and duplicated BUSCO	33.2%
Fragmented BUSCO	2%
Missing BUSCO	19.6%
Total BUSCO groups searched	954

### Functional annotation of transcriptome

Functional prediction and classification of the unigenes was conducted by searching the KOG and GO databases. For the KOG annotation, all the unigenes were annotated and classified into 26 functional categories (Fig. S1). The top three terms were: general function prediction only (2448, 20.48%); signal transduction mechanisms (1947, 16.29%); and posttranslational modification, protein turnover, chaperones (1015, 8.49%), respectively. However, a certain number of unigenes were assigned to unknown protein (843, 7.05%), due to the lack of available databases. GO is an international gene functional classification system that is utilized for functional categorization of DEGs [25]. Five thousand ninety-one unigenes were classified according to three major GO categories (Fig. S2). In the biological process category, “cellular process” was the most abundant GO term, while in the cellular component and molecular function categories, “cell part” and “catalytic activity” were the most enriched terms, respectively.

### Differential expression and functional analysis of assembled giant clam transcripts

**Fig. 2:**
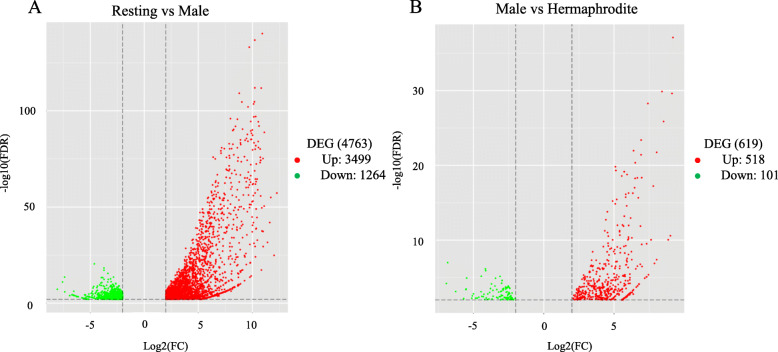
Volcano plot for gene differential expression in *T. squamosa* transcriptome. **a**: Resting vs Male; **b**: Male vs hermaphrodite. Unigenes with FDR ≤ 0.01 and ratio of FPKMs of the two samples ≥2 were considered to be differentially expressed genes. The red region shows significantly up-regulated genes, while the green region shows down-regulated genes

**Fig. 3:**
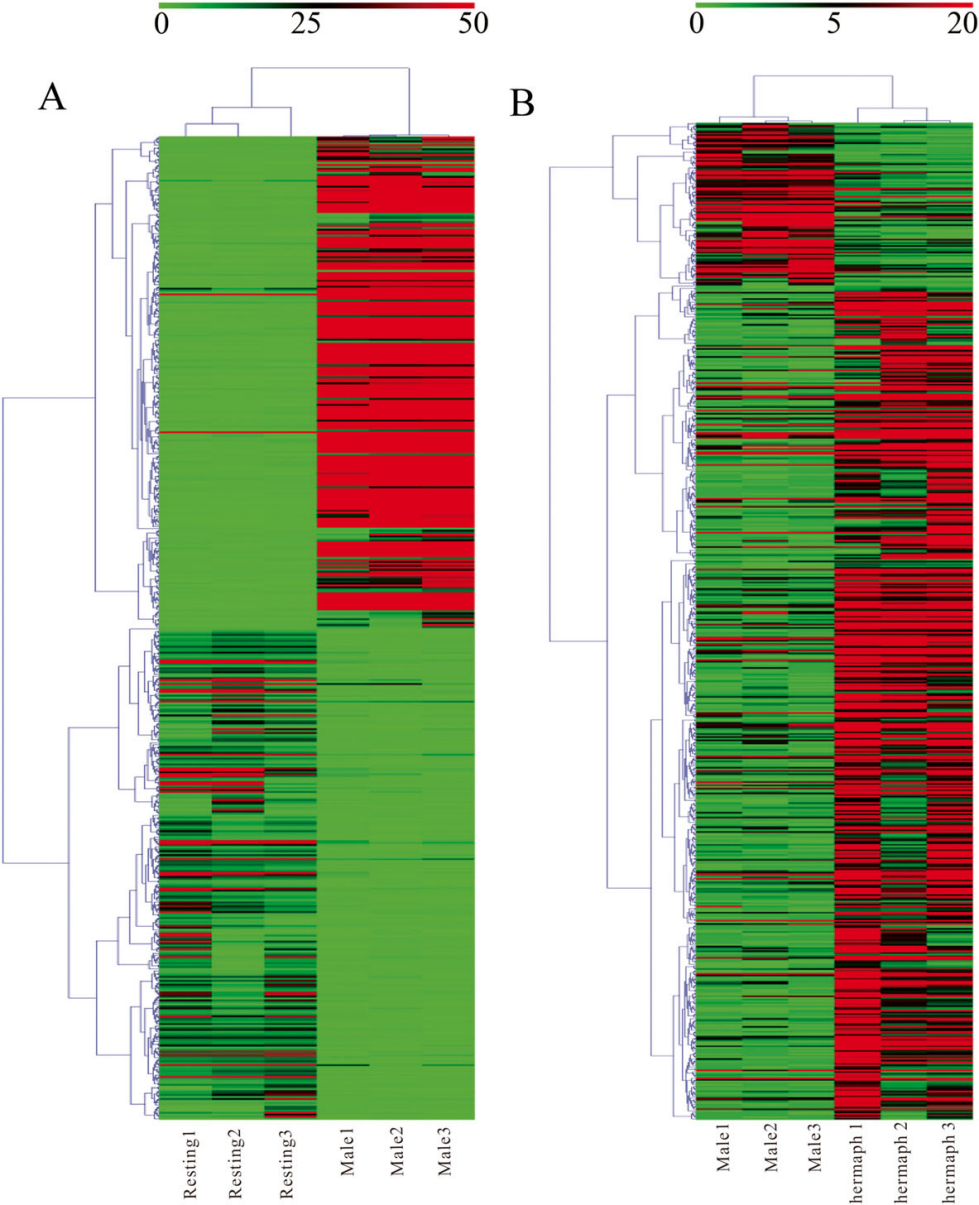
Hierarchical cluster analysis of selected differentially expressed genes (DEGs) of *T. squamosa*. **a**: Resting vs Male; **b**: Male vs hermaphrodite. Each column represents a sample, each row represents a gene, and each different color represents log2 fragments per kilobase of transcript per million mapped reads (FPKM) to indicate different expression levels. Green represents weakly expressed genes and red represents strongly expressed genes

To better survey the biological mechanism of gonad development, it is important to identify the genes which are differentially expressed between stages. To increase the accuracy of the measured expression levels for further analyses, data from 3 libraries derived from the biological replicates of each sample were mapped independently and later analyzed as biological replicates. And TPM (transcript per million) values were calculated based on the above data. Two groups (Resting versus Male, Male versus Hermaphrodite) were constructed to analyze DEGs using an FDR ≤ 0.01 and log_2_-Ratio ≥ 1. The former group (Resting versus Male) was identified to have 4763 DEGs, including 3499 up-regulated and 1264 down-regulated genes in males, while the latter (Male versus Hermaphrodite) had 619 DEGs, of which 518 were up-regulated and 101 were down-regulated in hermaphrodites (Table S3, S4). An overall view of the expression patterns between the two groups is shown in Fig. 2 (FDR ≤ 0.01 and log_2_-Ratio ≥ 1). Hierarchical cluster analysis showed that the clustering branch displayed the similarity of genes or samples, which conformed to the evaluation of biological replicates (Fig. 3).

**Fig. 4:**
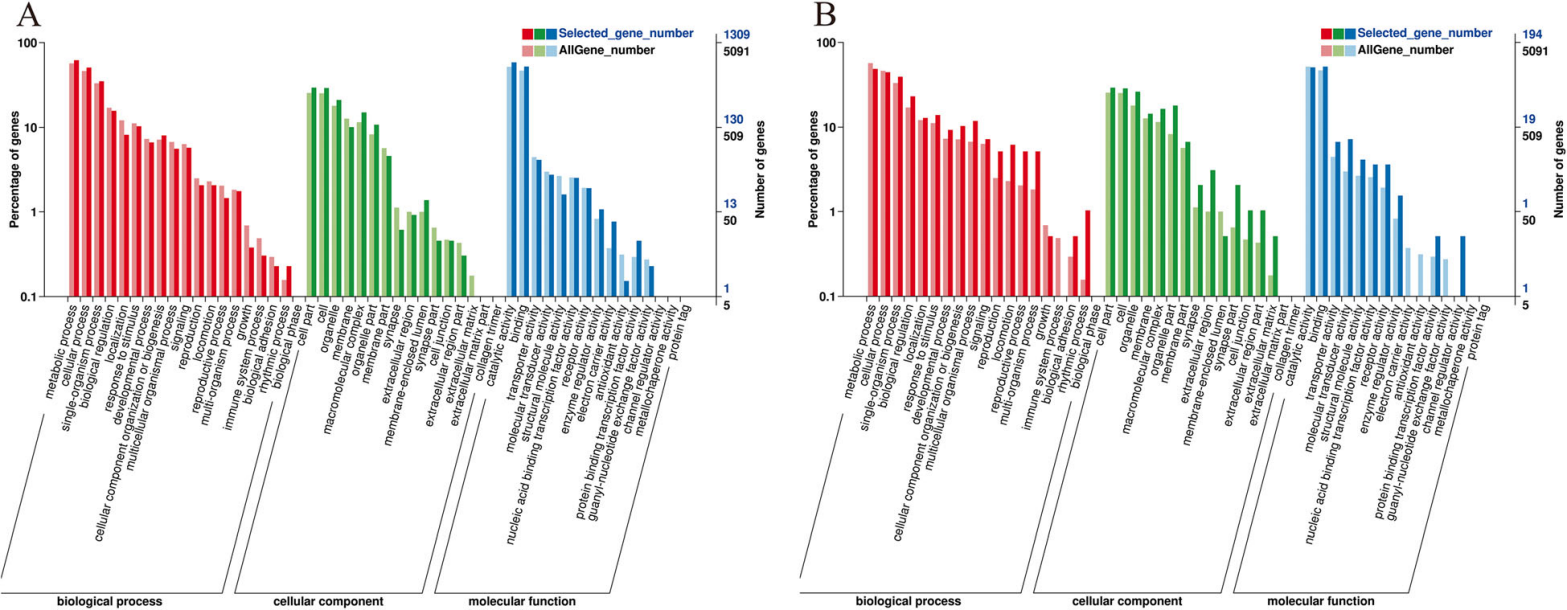
Gene Ontology (GO) functional classification of differentially expressed genes (DEGs) in *Tridacna squamosa*. **a**: Resting vs Male; **b**: Male vs hermaphrodite. The x-axis shows three terms and the y-axis shows the proportion of DEGs and unigenes corresponding to each subcategory. The red column represents annotation of all genes, while the blue column represents annotation of DEGs

Enrichment analysis in the molecular function, cellular component and biological process categories produced 613, 85 and 172 enriched GO-terms, respectively, for the Resting versus Male group, and 55, 12 and 14 for the Male versus Hermaphrodite group. (Table S5). The most-enriched GO-terms for the Resting versus Male group were “serine/threonine kinase activity” in the molecular function category, “chromosome” in the cellular component category, and “single-organism transport” in the biology process category. In the Male versus Hermaphrodite group, the most-enriched GO-terms were “lipid particle” and “membrane” in the cellular component category; “binding” and “signal transducer activity” in the molecular function category; and “oocyte maturation”, “activation of MAPKK activity” and “protein peptidyl-prolyl isomerization” in the biological process category (Fig. 4).

**Fig. 5:**
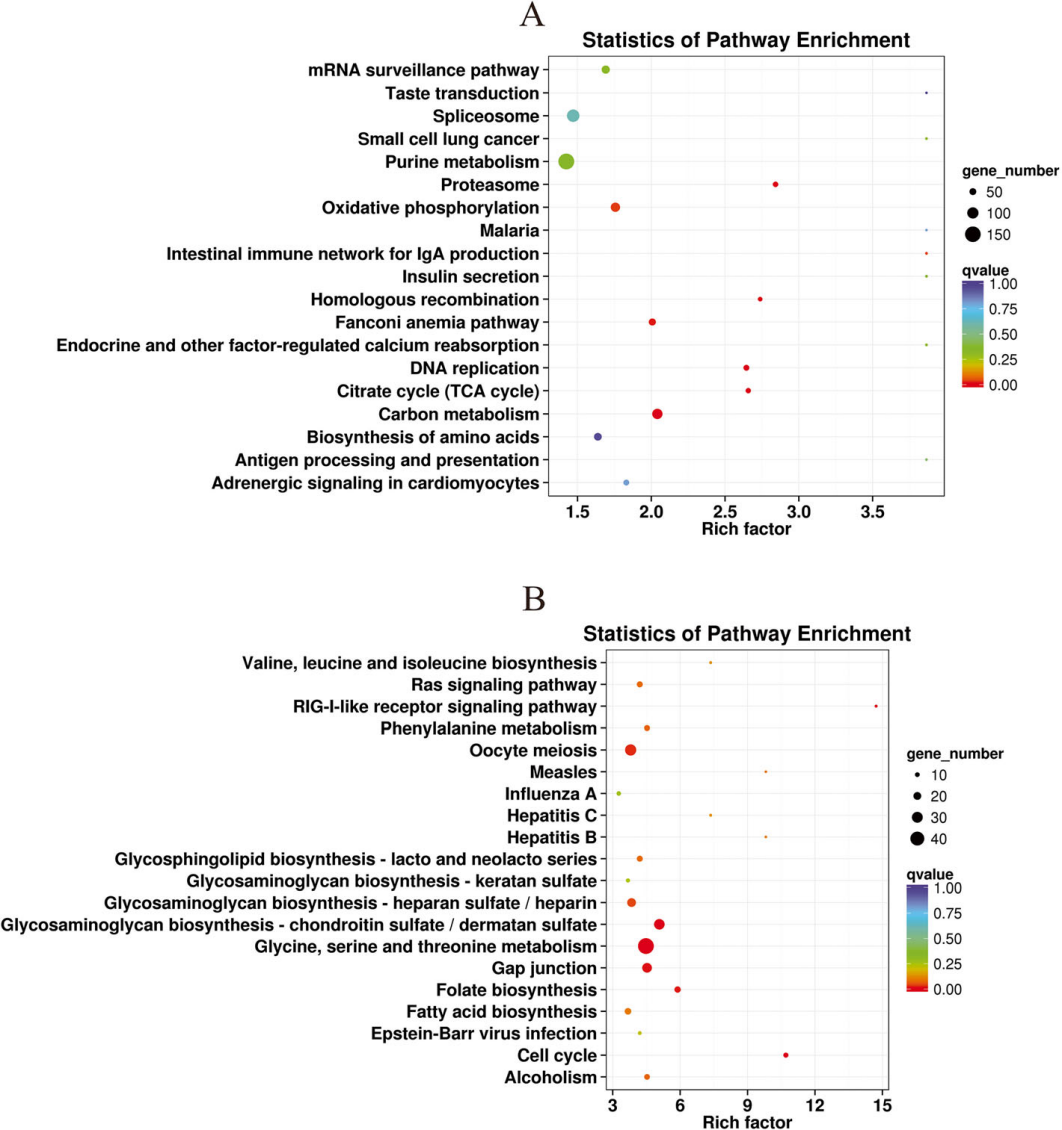
Statistics of Kyoto Encyclopedia of Genes and Genomes (KEGG) pathway enrichment analysis of the functional significance of DEGs. **a**: Resting vs Male; **b**: Male vs hermaphrodite. The abscissa is the enrichment factor, which increases the more significant the enrichment level of differentially expressed genes in the pathway. The ordinate is log10 (Q value), which increases with greater significance of differentially expressed genes in the pathway

To identify the biological pathways active in giant clam gonads, the differentially expressed genes were mapped to the reference canonical pathways in the KEGG database. Two hundred twenty-five and 112 signaling pathways were enriched in the Resting versus Male and Male versus Hermaphrodite groups, respectively. The top 20 most enriched KEGG pathways were showed by R packages in Fig. 5. In the Resting versus Male group, the five most-enriched pathways were “carbon metabolism” (ko01200), “oxidative phosphorylation” (ko00190), “purine metabolism” (ko00230), “citrate cycle” (TCA cycle; ko00020) and “proteasome” (ko03050). Additionally, three of the top 20 most-enriched pathways, “adrenergic signaling in cardiomyocytes” (ko04261), “insulin secretion” (ko04911) and “endocrine and other factor-regulated calcium reabsorption” (ko04961), play important roles in cellular functions such as proliferation, apoptosis, differentiation and migration, indicating the involvement of these pathways in the developmental process of spermatogenesis. For the Male versus Hermaphrodite group, the five most enriched pathways were “cell cycle” (ko04110), “glycine, serine and threonine metabolism” (ko00260), “RIG-I-like receptor signaling pathway” (ko04622), “glycosaminoglycan biosynthesis-chondroitin sulfate/dermatan sulfate” (ko00532) and “measles” (ko05162). Furthermore, several signaling pathways well-documented to be essential in gonadal development and maturation were found, including “oocyte meiosis” (ko04114), “ras signaling pathway” (ko04014), and “phenylalanine metabolism” (ko00360).

### Identification of genes involved in the regulation of gonad development

By analyzing the overall gene expression profiles of gonads, at least 31 genes involved in spermatogenesis were identified in the male group, including doublesex- and mab-3-related transcription factor, transcription factor Sox-8, sperm surface protein 17, sex determining protein Fem-1, TSSK4 and other potential candidates (Table 3). More than 40 genes, including both spermatogenesis (SPATA17, SOX8, SP17, SMKX, testis-specific serine/threonine kinases 4 and Sperm-associated antigen 8) and oogenesis genes (Zona pellucida, vitellogenin, 5-hydroxytryptamine receptor, Forkhead Box L2, vitellogenin receptor, and transcriptional regulator ATRX), were found to be responsible for the maintenance of hermaphrodite giant clams (Table 4). Identification of these essential genes and their regulatory mechanisms provides new understanding about the complex processes of reproduction and development. The information gained about these genes can be used to improve giant clam aquaculture.

**Table 3:** List of candidate genes significantly expressed in male gonads of giant clam

Seq id	Gene	Length	log2fold	Function
c140096.graph_c0	TSSK4	1466	10.98	Testis-specific serine/threonine-protein kinase 4
c233813.graph_c0	TSSK1	2750	10.23	Testis-specific serine/threonine-protein kinase 1
c233643.graph_c0	TSSK5	2759	10.02	Testis-specific serine/threonine-protein kinase 5
c139205.graph_c0	MARCH3	1049	10.01	E3 ubiquitin-protein ligase MARCH3
c192024.graph_c0	SP	639	9.783	Sperm-specific protein
c243537.graph_c0	SOX8	2521	8.48	Transcription factor Sox-8
c239972.graph_c0	SP17	3260	8.43	Sperm surface protein Sp17
c215109.graph_c1	TSS	1575	7.57	Testis-specific serine
c242073.graph_c0	SMKX	1941	7.37	Sperm motility kinase X
c245805.graph_c0	WEEL	3606	6.42	Mitosis inhibitor protein kinase wee1
c244841.graph_c2	SP	1962	6.38	sperm protein
c223469.graph_c1	RSP5	1087	6.20	E3 ubiquitin-protein ligase RSP5
c238287.graph_c0	CB3	1990	5.97	cyclin B3
c226708.graph_c0	SP17	1558	5.89	Sperm surface protein Sp17
c230037.graph_c0	SPATA17	1524	5.72	Spermatogenesis-associated protein 17
c248751.graph_c0	SPEF2	7300	5.30	Sperm flagellar protein 2
c234750.graph_c0	CYCA	1997	5.10	G2/mitotic-specific cyclin-A
c236288.graph_c0	SPATA7	2109	4.88	Spermatogenesis-associated protein 7
c244022.graph_c0	CYCF	3199	4.78	G2/mitotic-specific cyclin-F
c243072.graph_c0	CYCB	3237	4.54	cyclin B
c242769.graph_c0	SPAG6	1940	4.52	Sperm-associated antigen 6
c241588.graph_c0	SPATA6	4146	4.12	Spermatogenesis-associated protein 6
c240907.graph_c1	THEP	2056	3.84	testicular haploid expressed protein
c236645.graph_c0	CDC20L	2045	3.49	Cell division cycle protein 20-like protein
c229404.graph_c0	SP	935	3.47	sperm protein
c221360.graph_c0	CDCA3	912	3.03	Cell division cycle-associated protein 3
c205982.graph_c0	CKS1B	1225	2.91	Cyclin-dependent kinases regulatory subunit 1
c214859.graph_c0	FEM-1	1162	2.83	sex determining protein Fem-1 protein
c242650.graph_c0	CYP356A1	2436	2.26	Cytochrome P450
c246248.graph_c0	PIWI1	4389	1.88	Piwi-like protein 1
c264870.graph_c0	DMRT	459	5.65	Double sex/Male-abnormal-3 Related Transcription factor

**Table 4:** Candidate genes significantly expressed in hermaphroditic gonads of giant clam

Seq id	Gene	Length	Log2fold	Function
c234386.graph_c0	ZP	1486	9.19	Zona pellucida-like protein
c242366.graph_c0	ZP	601	9.13	Zona pellucida-like protein
c235100.graph_c0	FOXI1	1474	8.86	Forkhead box protein I1
c243164.graph_c0	ZP	3559	8.41	Zona pellucida-like protein
c236716.graph_c0	ZP	1706	6.93	Zona pellucida-like domain
c233951.graph_c0	ZP	1235	6.92	Zona pellucida-like protein
c238909.graph_c0	ZP	2235	6.66	Zona pellucida-like protein
c248656.graph_c0	VI	8276	6.51	vitellogenin
c235080.graph_c0	ZP	1416	6.34	Zona pellucida-like protein
c233717.graph_c0	CYCB	1503	5.07	G2/mitotic-specific cyclin-B
c179388.graph_c1	P62	693	5.24	Mitotic apparatus protein p62
c233717.graph_c0	CYCB	1503	5.07	G2/mitotic-specific cyclin-B
c236606.graph_c0	FOXN2	1351	4.56	Forkhead box protein N2
c243687.graph_c0	LHX6	1031	4.51	Lhx6/8 LIM homeobox protein
c231661.graph_c0	5HTRB2	1516	4.34	5-hydroxytryptamine receptor 2B
c240984.graph_c0	DRD2	2222	3.31	D(2) dopamine receptor
c232796.graph_c0	FOXL2	1421	3.09	Forkhead box protein L2
c247556.graph_c0	CNGB1	3589	2.76	Cyclic nucleotide-gated cation channel beta-1
c245914.graph_c0	5HTR	2723	3.26	5-hydroxytryptamine receptor
c227931.graph_c0	VGR	960	4.64	vitellogenin receptor
c248300.graph_c0	ATRX	6575	3.12	Transcriptional regulator ATRX homolog
c140096.graph_c0	TSSK4	1466	10.89	Testis-specific serine protein kinase 4
c192024.graph_c0	SP	639	9.42	Sperm-specific protein
c239972.graph_c0	SP17	3260	8.19	Sperm surface protein Sp17
c243537.graph_c0	SOX8	2521	7.33	Transcription factor Sox-8
c242073.graph_c0	SMKX	1941	6.93	Sperm motility kinase X
c230037.graph_c0	SPATA17	1524	5.10	Spermatogenesis-associated protein 17
c244841.graph_c2	SP	1962	5.06	sperm protein
c188535.graph_c0	SPAA8	870	4.71	Sperm-associated antigen 8

### Validation of differentially expressed genes using qRT-PCR

**Fig. 6:**
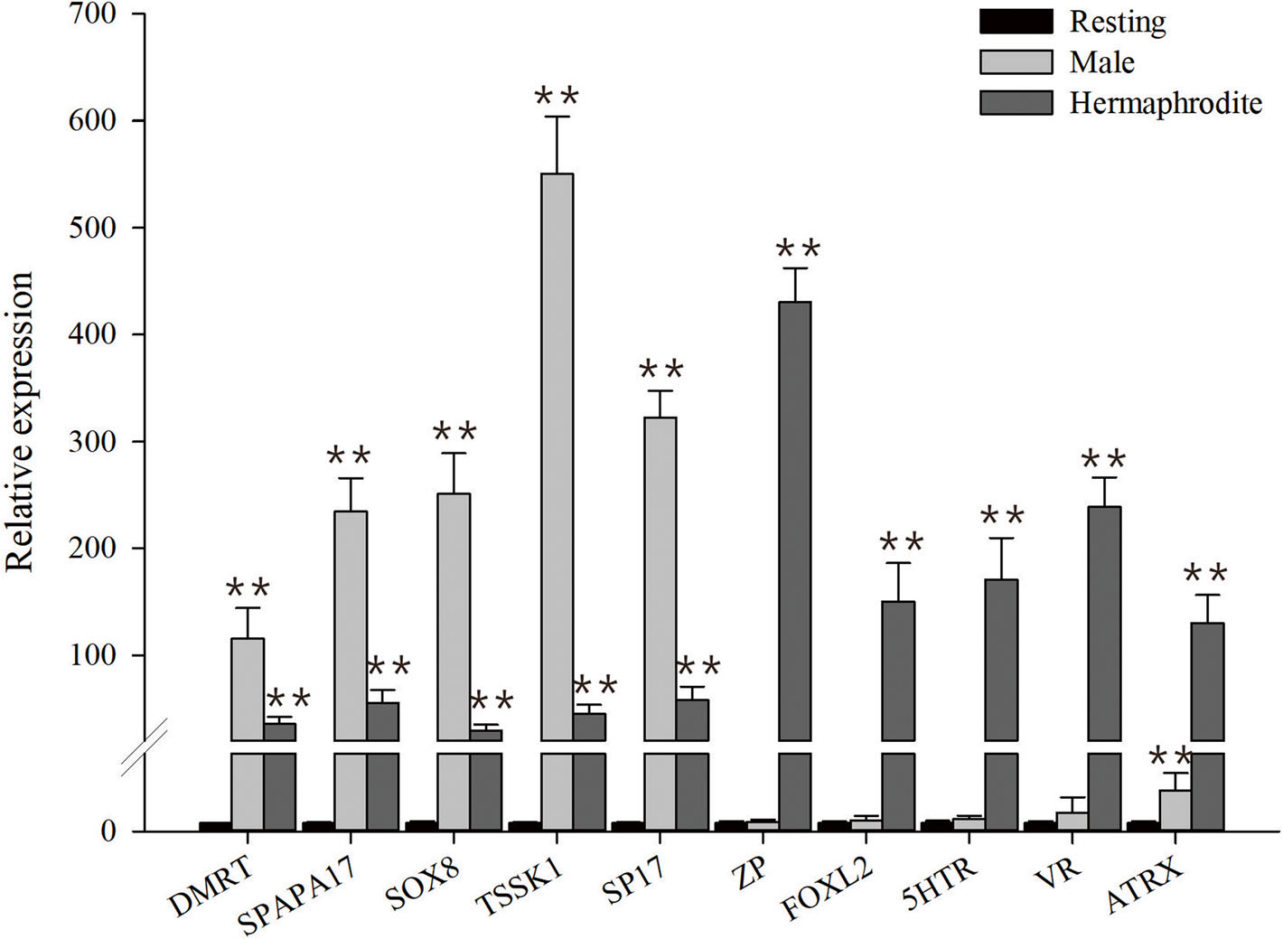
RT-PCR analysis of differential gene expression in different gonad development stages of *T. squamosa*. The relative mRNA expression of 10 genes was calculated using the2^-∆∆Ct^ method, with EF1α as a reference gene. The data are presented as the mean relative expression ± standard deviation (SD) of three replicate real-time reactions from pooled tissue samples (*N* = 5). Significant differences are indicated: **p < 0.01

To validate the expression levels of DEGs identified by RNA-Seq in gonads, we randomly selected 10 DEGs related to sex-differentiation (DMRT, SPAPA17, SOX8, TAAK1, SP17, ZP, FOXL2, 5HTR, VGR, ATRX) for qRT-PCR validation. Expression of DMRT, SPAPA17, SOX8, TSSK1, and SP17 was higher in testes, whereas ZP, FOXL2, 5HTR, VGR, ATRX were found to be elevated in ovaries. Comparison of the transcriptome data from RNA-Seq with the qRT-PCR results from seven selected differentially expressed genes revealed that they were consistent with each other at these gonad developmental stages (Fig. 6). These results reiterate the differential gene expression pattern observed in gonadal transcriptome analysis.

## Discussion

Gonad development is a very complex and critical process which begins before sexual differentiation. During this process, many genes cause the gonad to differentiate into either a testis or ovary and, subsequently, cause the development of a male, female, or hermaphroditic phenotype [26–30]. Giant clams are protandrous hermaphrodites [31]. Their sequential sexual development begins in the juvenile stage with no visible gonads and progresses to the development of testes, which is followed later by ovary development, resulting in hermaphroditic individuals [3]. Recent research on the sex determination mechanisms and sex-related genes of mollusks has made considerable progress with the advancement of next-generation sequencing technology. However, research efforts have mainly focused on dioecious mollsuks such as *Haliotis rufescens* (Myosho et al., 2012), *Chlamys nobilis* [32] *Patinopecten yessoensis* [33] *Haliotis discus discus* [34] *Crassostrea hongkongensis* [35] *Mytilus edulis* [36] and *Crassostrea gigas* [37]; studies on hermaphroditic mollusks such as giant clams are extremely scarce. Thus, it’s vital to identify genes that are involved in the gonadal development of hermaphroditic animals. Here, we proposed to unravel some molecular mechanisms and genes involved in gonad development and gametogenesis of a tropical marine hermaphrodite mollusk, *T. squamosa*, using Illumina-based RNAseq.

### Annotation of giant clam gonad transcriptome

To obtain a gonadal expression profile from the giant clam, 9 samples of gonads in different reproductive stages were sequenced using an Illumina HiSeq2500 high-throughput sequencing platform. From these, a total of 124,564 transcripts (N50 = 1488) and 95,408 unigenes (N50 = 1143) were identified. On average, the statistics for the de novo assemblies are similar to those for other transcriptomes of other species [38–40]. Because no reference genome exists for giant clams, the high-quality reads from the nine libraries were combined and assembled into a reference transcriptome using Trinity software with default parameter settings [41]. Annotation results showed that more than half (72.72%) of unigenes could not be assigned to known genes, making it difficult for transcriptomes annotation, which is consistent with previous studies in the Pacific oyster (24.42% annotation) [42], and *Laternula elliptica* (16.93% annotation rate) [43]. The poor annotation efficiency could be due to insufficient sequences in public databases for phylogenetically closely related species. Some of the unannotated sequences may be untranslated regions, non-coding RNAs, small RNAs, or short sequences which do not contain known protein domains [44]. In addition, 4763 and 619 DEGs were identified in Resting versus Male and Male versus Hermaphrodite group, respectively. Interestingly, the number of DEGs in Resting versus Male group was much greater than that in Male versus Hermaphrodite group. This may be due to the fact that many researchers are mainly concerned with the reproductive phenotype of male bivalves. Consequently, male bivalves have much more genetic information than females.

Although several bivalve species (e.g. oysters, scallops, mussels, etc.) have been the target of transcriptome studies, so far these have not been applied to giant clams. This study identifies the first transcriptome relating to gonadal expression profile in giant clams, which will provide abundant information for the future study of giant clams.

Among the biological processes, it is noteworthy that “reproduction” (GO: 0000003) and “reproductive process” (GO: 0022414), which relate to reproduction and gonadal development, were also enriched. This was similar to results found in other mollusks, including *Patinopecten yessoensis* [45], *Reishia clavigera* [46], *C. farreri* [47]. For example, in *P. yessoensis*, the most-enriched terms were “cellular process” in biology process, “cell” and “cell part” in cellular component and “cellular component” in molecular function. This demonstrates the similarity of mRNA composition in mollusks.

The Kyoto Encyclopedia of Genes and Genomes (KEGG) contains functional information on metabolic pathways or regulatory networks of genes and interacting molecules in cells, which is very useful in the study of the complex biological behaviors of genes [48]. In model organisms, it has been reported that the oocyte meiosis pathway plays a vital role in ovary development [49, 50]. The Ras proteins are GTPases that function as molecular switches for signaling pathways regulating cell proliferation, survival, growth, migration, differentiation or cytoskeletal dynamism [51]. Hydroxylation of phenylalanine into tyrosine is essential for oogenesis and oocyte maturation in the *Anopheles gambiae* [52]. These pathways were assigned to the KEGG categories of “signal transduction” and “endocrine system”, indicating the significance of signal transduction systems and endocrine regulation in ovarian differentiation and maintenance in giant clams. The GO and KEGG annotations were helpful for identifying potential genes with specific functions from a large-scale transcriptome database, and provided a substantial resource for studying significant processes, functions and pathways during gonadal development in giant clam.

### The candidate genes related to male gonad development of giant clam

The DEGs that were identified in the Resting versus Male comparison included several well established genes involved in gonadal development and gametogenesis, such as the testis-specific serine/threonine kinases 1 (TSSK1), TSSK4, TSSK5, Doublesex- and mab-3-related transcription factor 1 (DMRT1), SOX, Sperm surface protein 17 (SP17), Sperm-specific protein, and others. Below, several well documented and important candidate genes are listed in more detail.

Doublesex- and mab-3-related transcription factor (DMRT) is a sex determining gene that is essential for the maintenance of male-specified germ cells and testis differentiation [53, 54]. At present, eight members of the family (DMRT1–8) have been reported in vertebrates [55]. In mollusks, orthologs of DMRT have been characterized from the oysters *C. gigas* [56], *P. martensii* [14] and *Pinctada fucata* [57]. In our study, DMRT1 was indentified in giant clams, with higher expression in the male gonad than in resting and hermaphroditic gonads. The results of qRT-PCR were also consistent with the RNA-Seq results. These results reveal that DMRT may be an important factor for sex determination and differentiation of *T. squamosa*.

The Sox transcription factors exert essential functions that regulate male sex determination and testicular differentiation [58]. The Sox family has more than 20 homologues across different species. The transcription factor Sox9 is the direct target of Sry and plays a pivotal role in the male gonadal development of many vertebrates [59]. Recently, Sox9 as a critical factor in sex determination and differentiation was confirmed in a marine mollusk [12]. In *Haliotis asinina*, SOXB and SOXC were detected in the cerebral and pleuropedal ganglia and SOXB was found to participate in neural structure formation in the limpet *Patella vulgata* [60, 61]. In the cephalopod *Sepia officinalis*, three members of SOX from group B and E were identified, which showed different expression patterns in early embryogenesis and vasculogenesis [62]. A gene closely related to Sox30 in vertebrates has been reported in *C. gigas* and was exclusively expressed in the testis [37]. In *C. farreri*, the expression levels of *SoxB2* were similar in male and female gonads at different developmental stages of reproduction [63]. In the present study, we identified a SOX8 gene and the results showed that its expression levels were significantly different between males and restings. Although the detailed function of SOX8 is unknown, we speculate that Sox8 is likely to replace the function of Sry in testis determination in the giant clam.

The testis-specific serine/threonine kinase (TSSK) family may have an important role in sperm differentiation in the testis and/or fertilization. Six members of this family have been reported: TSSK1-TSSK6 [64]. These protein kinases belong to the 5′ adenosine monophosphate-activated protein kinase (AMPK) family. However, it is not yet known whether TSSK5 has all the domains needed to be an active kinase [65]. In the mouse, the male-biased genes TSSK1 and TSSK5 are essential for stages of spermatogenesis, and knockout of TSSK1 resulted in deficiencies in spermatogonial or spermatocytic apoptosis [66]. TSSK4 knockout male mice exhibited a subfertile phenotype due to seriously decreased sperm motility [67]. TSSK6 deletion resulted in an infertile male phenotype caused by certain morphological defects in the sperm [68]. In our trancriptome, TSSK1, TSSK4, and TSSK5 were identified and exhibited high levels of expression in male gonads. These results are similar to those found in *P. yessonsis*, suggesting an important role of TSSK in giant clam spermatogenesis [69]. In addition, the present study also found significantly higher expression of SP17 and Sperm Specific Protein in male than in resting and hermaphroditic clams, and the expression difference might be associated with spermatogenesis. In bovine sperm, SP17 had been found to be essential for spermatogenesis and played significant roles in fertilization events, such as membrane remodeling, transport, protection and function [70]. However, the role of SP17 in *T. squamosa* spermatogenesis remains further investigation.

### The candidate genes facilitating to hermaphroditic gonad development of giant clam

The ovary is the female reproductive organ and is responsible for estrogen synthesis and oogenesis. Oogenesis consists of sequential and continuous changes in the cytoplasmic and nuclear content of maturing oocytes, which are regulated by a series of genes [71]. In this study, sevaral unigenes related to oogenesis were selected from the giant clam transcriptome data and verified by qPCR, including zona pellucida protein (ZP), Forkhead Box L2 (Foxl2), Vitellogenin, Vitellogenin receptor (VgR), 5-hydroxytryptamine (5-HT), and 5-hydroxytryptamine receptor (5-HTR).

The zona pellucida protein (ZP), also known as the egg coat protein, is involved in oocyte and gamete development and mediates sperm-oocyte binding, as well as sperm penetration [72]. In fish, the expression of ZP gene mRNA is significantly increased during oogenesis, especially at the previtellogenic stage, when the expression level is higher than at undeveloped stages [73]. In *Acipenser sinensis*, two ZP proteins (AsZPAX and AsZPB) were identified in the developing oocytes, which will shed light on the regulatory mechanism of egg envelope formation and fertilization process in this fish [74]. Previous studies have shown that the ZP2 gene played a key role in the early formation of the oocyte envelope and ZP3 was a major class of female-specific reproduction-related molecules [75]. Recent evidence indicates that at least nine ZP domain proteins are prominent components of the egg coat of marine gastropod abalone. In this study, a large number of unigenes in hermaphroditic giant clam gonads were annotated with the various domains of the VEZP protein and showed higher expression levels in the ovary than in the testis, suggesting that these oocyte-specific genes may also play important roles in oocyte maintenance and reproduction.

FOXL2, originally identified in *Drosophila*, is an essential gene controlling the differentiation and development of the ovary in vertebrates [76, 77]. In vertebrates, FOXL2 localizes to the granulosa cells and the early ovarian stroma, and knockout of this gene causes disordered ovarian follicular formation and partial ovary-to-testis sex reversal [78]. In *Crassostrea gigas*, Cg-FOXL2 is significantly more expressed in mature females than in mature males, indicating its role in vitellogenesis or female sex determination [79]. In *C. farreri*, Cf-FOXL2 is specifically expressed in the ovary during gonadogenesis and adult gonadal development cycle, which suggests that it can be used as a female marker and a key gene in the study of ovary identification [80]. In this study, Foxl2 was predominantly expressed in the ovary, suggesting a role in ovarian differentiation and development in giant clams. A FOXN2 and FOXI1 gene, belonging to the FKH family, showed significant differences in expression between males and females, suggesting that they also may be involved in ovarian determination, although the precise mechanisms remain unclear.

Other genes involved in the formation and maintenance of oocytes were also found in the gonad transcriptome of the giant clams in this study. As observed in many model organisms, oocyte maturation depends on massive production of the egg yolk precursor protein, vitellogenin (VG). Vitellogenin receptor (VGR) is involved in Vg uptake by oocytes and plays a critical role in egg development [81]. In the current study, we found high occurrence of Vg and VgR in the ovaries, and RT-PCR demonstrated that the expression level in hermaphroditic gonads was significantly higher than in male gonads, providing some clues to further elucidate its function in ovary development in giant clams. 5-hydroxytryptamine (5-HT) has been found to play a variety of biological roles in gonad maturation and sequential spawning [82, 83]. It acts through 5-HT receptors, a superfamily of G-protein-coupled receptors, to modulate oocyte development and maturation in vertebrates and invertebrates [84, 85]. In the present study, both the 5-HT and 5-HT receptor were significantly high expression in hermaphroditic gonads, which indicated its poetical role in ovary development.

In addition to these female-related genes, our study observed significantly higher expression of TSSK4, spermatogenesis-associated protein 17 (SPAG17), SPAG8, sperm motility kinase X (SMKX) and SP17 in hermaphroditic gonads than in resting gonads, and the expression difference might be associated with the maintenance of spermatogenesis. The spermatogenesis-associated gene (SPATA), a testis-specific gene, played an important role in maintaining the physiological function of germ cells involved in the regulation of apoptosis during spermatogenesis [86]. SPATA4 has previously been confirmed to be specifically expressed in the testes of animals ranging from mammals to birds [87, 88]. SPATA6 has been shown to be a critical protein in either the assembly or structural integrity of the sperm tail axoneme [89, 90]. TSSK4, SPAG17, SPAG8, SMKX and SP17 have been shown to be essential for spermatogenesis [39]. All these results indicate that both spermatogenesis- and oogenesis-related genes were simultaneously highly expressed in the hermaphroditic gonad to maintain the hermaphroditism of these giant clams.

## Conclusion

Taken together, this work represents the first attempt to use RNA-seq technology to identify gonad development and gametogenesis genes in resting, male and hermaphroditic giant clam gonads. Based on the comparative transcriptome analysis, we obtained 95,408 unigenes, which were assigned to biological processes and functions after annotation in GO, KOG and KEGG databases. A significant number of gonad-related functional genes and sex-related biological pathways were found and many of them were involved in gonad development. These results provide novel insights into the genetic mechanism of giant clam sex determination and also facilitate future research into aquaculture breeding of giant clams.

## Methods

### Ethics statement

All procedures during this study were conducted in accordance with the guidelines of the South China Sea Institute of Chinese Academy of Sciences Animal Care and Use Committee.

### Experimental giant clams and samples collection

Twenty five Giant clams were collected from Xisha island China on December 10th 2016 for scientific purposes. Then, they were acclimated with aerated sand-filtered seawater in the hatchery in Sanya at 28 ± 2 °C before the experiment. The resting gonad samples were collected on 12 December, 2017. The male and hermaphrodite gonad samples were collected on 78 days and 123 days, respectively, after the first sampling. Gonad tissues were sampled for RNA extraction and fixed for histology analysis. The gonad tissues were taken using a hypodermic needle through biopsy, immediately immersed in the RNAlater (Ambion, Austin, TX, USA), and stored at − 80 °C until RNA preparation or fixation in Bouin’s solution for histological analysis. Fixed gonad tissues were dehydrated with serial solutions of ethanol, and embedded in paraffin. The standard 6-μm sections were stained following the standard haematoxylin-eosin staining protocol for histological analysis. Resting-stage gonads were identified by the absence of sperm and oocytes. Male-stage gonads were identified by the presence of well-developed sperm. Hermaphrodite-stage gonads were identified by the presence of both sperm and oocytes.

### RNA isolation, cDNA library construction and sequencing

Total RNA was extracted from gonad suspensions in RNA-free microcentrifuge tubes containing 1 mL TRIzol Reagent (Invitrogen, Carlsbad, CA) per 100 mg of tissue, according to the manufacturer’s instructions. RNA concentrations were measured using a Nanodrop 2000c Spectrophotometer (Thermo Scientific, USA) and the quality of the RNA was analyzed with Bioanalyzer 2100.

RNA-Seq library preparation and sequencing were carried out for three resting, three male and three Hermaphrodite gonads using Illumina TrueSeq RNA Sample Preparation Kit according to the manufacturer’s instructions. The mRNA was isolated from total RNA using Sera-mag magnetic oligo-(dT) beads (Illumina), and fragmented into short fragments with fragmentation buffer. The fragmented mRNA was then subjected to cDNA synthesis using random hexamer primer. After end repair and addition of 3’dA overhangs, the double-stranded cDNA was ligated into the Illumina TruSeq adaptors and enriched by polymerase chain reaction. The libraries were subjected to 150 bp paired-end sequencing on the Illumina HiSeq 2500. All the obtained data have been submitted to the NCBI Sequence Read Archive under under the BioProject PRJNA598738.

### Transcriptome assembly and annotation

Before assembly, the raw Illumina reads were converted to FASTAQ format, and trimmed to remove the adaptor sequences, duplicated sequences, ambiguous reads and low-quality reads using the tools from FASTX-Toolkit. Due to the lack of genome information on *T. squamosa*, the clean reads from nine libraries were mixed together as a reference database, and de novo assembled using Trinity v.2.4.0 with default parameter settings [41]. CD-HIT (version 4.6.5) with the setting of “-c 0.95” was used to remove transcripts whose sequence similarity exceeded 95% [91]. The resulting sequences were called unigenes. Analysis of assembly completeness was performed using BUSCO v3 obtain the percentage of single-copy orthologs represented in the metazoa_odb9 dataset [92].

Open reading frames (ORFs) of transcript and unigene sequences were predicted by the Trans-Decoder package (http://transdecoder.sourceforge.net/), with the minimum ORF length of 100 bp. For homology annotation, unigenes were subjected to analysis using public databases, including NCBI (http://www.ncbi.nlm.nih.gov/) non-redundant protein (NR), Swiss-Prot, PFAM, KOG, eggNOG, and COG databases with an E-value cutoff of 10^− 6^. Gene names and descriptions were assigned to each contig based on the BLASTX results. Afterwards, Blast2GO software was employed to obtain Gene Ontology (GO) annotations of transcripts with an E-value cutoff of 10^− 6^ and WEGO software was used to perform GO functional classification of all unigenes in order to understand the distribution of gene functions. Kyoto Encyclopedia of Genes and Genomes (KEGG) pathways were used to assemble transcripts with the KEGG Automatic Annotation Server (http://www.genome.jp/kegg/kaas/).

### Differential expressed genes and analysis of potential reproduction-related genes

The cleaned reads of each RNA-seq library were mapped to the assembled transcripts with Bowtie program [93]. Unique mapped reads, including paired-end reads for which only one part matched, were used to calculate the level of gene expression using the transcripts per kilobase million (TPM), which is able to eliminate the influence of different gene lengths and sequencing levels using RSEM [94]. The edgeR method was used to identify differentially expressed genes (DEGs) between two groups [95]. The threshold for the *p*-value was determined by the false-discovery rate (FDR). Unigenes with FDR ≤ 0.01 and fold change of the two samples ≥2 (genes for which FPKM < 1 were filtered) were considered to be differentially expressed genes in this study. Three biological replicates were designed to acquire more reliable DEGs. To further investigate these DEGs, GO and KEGG enrichment analyses were performed using the algorithm implemented in GO stats [96]. A *p* value ≤0.05 designated a significantly enriched gene set and pathway among transcriptomic profiles.

### Quantitative real-time PCR validation

Quantitative real-time PCR (qRT-PCR) was used to verify the reliability of our sequencing and analysis obtained from the transcriptome data. Ten unigenes were randomly selected to design primers and EF1α was used as an internal control gene. The qRT-PCR analysis was performed on a Lightcycler480 (Roche, Basel, Switzerland) using a SYBR Green MasterMix (SYBR Premix EX Taq™, TaKaRa), according to the manufacturer’s instructions. Primers used for qRT-PCR are listed in Table S1. Reactions were performed in triplicate, and the relative expression levels were calculated using the 2^-∆∆Ct^ method [97]. The Student’s t-test was conducted and significant differences were determined at a *p*-value of < 0.05 or < 0.01.

## Supplementary Information

**Additional file 1: Table S1.** Sequences of primers used in this study

**Additional file 2: Table S2.** Correlation analysis between different gonadal development groups.

**Additional file 3: Table S3.** The up- and down-regulated transcirpts bewteen Resting and Male libraries.

**Additional file 4: Table S4.** The up- and down-regulated transcirpts bewteen Male and hermaphrodite libraries.

**Additional file 5: Table S5.** Gene classification based on gene ontology (GO) for DEGs in Resting vs Male and Male vs hermaphrodite group

**Additional file 6: Figure S1.** Clusters of orthologous group (COG) function classifications of *T. squamosa*. The x-axis shows 25 categories, while the y-axis shows the number of DEGs corresponding to each category.

**Additional file 7: Figure S2.** GO distributions of *T. squamosa* transcriptomes.

## Data Availability

All data supporting the conclusions of this article are provided within the article and its supplementary files. All RNA-Seq data are available in the NCBI Sequence Read Archive database under BioProject accession number PRJNA598738 (www.ncbi.nlm.nih.gov/bioproject/PRJNA598738).

## Abbreviations

cDNA: Complementary deoxyribonucleic acid
GO: Gene ontology
PCR: Polymerase chain reaction
ORF: Open reading frame
DEGs: Differentially expressed genes
KEGG: Kyoto Encyclopedia of Genes and Genomes (KEGG)
TPM: Transcript per million
TSSK: Testis-specific serine/threonine kinases
SP17: Sperm surface protein 17
DMRT: Doublesex- and mab-3-related transcription factor
ZP: Zona pellucida protein
FOXL2: Forkhead Box L2
5HTR: 5-hydroxytryptamine, 5-hydroxytryptamine receptor
CITES: Endangered Species of Wild Fauna and Flora
IUCN: International Union for Conservation of Nature
SOX8: Transcription factor Sox-8
SMKX: Sperm motility kinase X
SPATA7: Spermatogenesis-associated protein 7
FEM-1: Sex determining protein Fem-1 protein
ATRX: Transcriptional regulator ATRX homolog
SPAA8: Sperm-associated antigen 8
VGR: Vitellogenin receptor

## References

[1] Lesoway MP, Henry JQ (2019). Sex determination, sexual development, and sex change in slipper snails. Results Probl Cell Differ.

[2] Neo ML, Eckman W, Vicentuan K, Teo SLM, Todd PA (2015). The ecological significance of giant clams in coral reef ecosystems. Biol Conserv.

[3] Soo P, Todd PA (2014). The behaviour of giant clams (Bivalvia: Cardiidae: Tridacninae). Mar Biol.

[4] Pandolfi JM, Bradbury RH, Sala E, Hughes TP, Bjorndal KA, Cooke RG, McArdle D, McClenachan L, Newman MJH, Paredes G (2003). Global trajectories of the long-term decline of coral reef ecosystems. Science.

[5] Benzie JAH, Williams ST (1996). Limitations in the genetic variation of hatchery produced batches of the giant clam. Tridacna Gigas Aquacult.

[6] BENZIE JAH (2015). Genetic structure of coral reef organisms: ghosts of dispersal Past1. Am Zool.

[7] Watson SA (2015). Giant Clams and Rising CO2: Light May Ameliorate Effects of Ocean Acidification on a Solar-Powered Animal. PLoS One..

[8] Lee SLJ, Horsfield JA, Black MA, Rutherford K, Fisher A, Gemmell NJ (2017). Histological and transcriptomic effects of 17alpha-methyltestosterone on zebrafish gonad development. BMC Genomics.

[9] Wang J, Tian GG, Zheng ZX, Li B, Xing QH, Wu J (2019). Comprehensive transcriptomic analysis of mouse gonadal development involving sexual differentiation, meiosis and gametogenesis. Biol Proced Online..

[10] Gonzalez-Castellano I, Manfrin C, Pallavicini A, Martinez-Lage A (2019). De novo gonad transcriptome analysis of the common littoral shrimp Palaemon serratus: novel insights into sex-related genes. BMC Genomics..

[11] Naimi A, Martinez AS, Specq ML, Diss B, Mathieu M, Sourdaine P (2009). Molecular cloning and gene expression of cg-Foxl2 during the development and the adult gametogenetic cycle in the oyster Crassostrea gigas. Comp Biochemist Physiol Part B Biochemist Mol Biol.

[12] Santerre C, Sourdaine P, Adeline B, Martinez AS (2014). Cg-SoxE and cg-beta-catenin, two new potential actors of the sex-determining pathway in a hermaphrodite lophotrochozoan, the Pacific oyster Crassostrea gigas. Comp Biochem Physiol A Mol Integr Physiol.

[13] Shi Y, Wang Q, He MX (2014). Molecular identification of dmrt2 and dmrt5 and effect of sex steroids on their expressions in Chlamys nobilis. Aquaculture.

[14] Yu F-F, Wang M-F, Zhou L, Gui J-F, Yu X-Y: Molecular cloning and expression characterization of <i>Dmrt2</i> in Akoya pearl oysters, <i>Pinctada martensii</i>. J Shellfish Res 2011, 30(2):247–254, 248.

[15] Milani L, Ghiselli F, Iannello M, Passamonti M (2014). Evidence for somatic transcription of male-transmitted mitochondrial genome in the DUI species Ruditapes philippinarum (Bivalvia: Veneridae). Curr Genet.

[16] Bettinazzi S, Plazzi F, Passamonti M (2016). The complete female- and male-transmitted mitochondrial genome of Meretrix lamarckii. PLoS One.

[17] Fitt WK, Trench RK (1981). Spawning, development, and Acquisition of Zooxanthellae by Tridacna-Squamosa (Mollusca, Bivalvia). Biol Bull.

[18] Yue C, Li Q, Yu H (2018). Gonad Transcriptome analysis of the Pacific oyster Crassostrea gigas identifies potential genes regulating the sex determination and differentiation process. Mar Biotechnol.

[19] Piprek RP, Damulewicz M, Tassan JP, Kloc M, Kubiak JZ (2019). Transcriptome profiling reveals male- and female-specific gene expression pattern and novel gene candidates for the control of sex determination and gonad development in Xenopus laevis. Dev Genes Evol.

[20] Tao W, Yuan J, Zhou L, Sun L, Sun Y, Yang S, Li M, Zeng S, Huang B, Wang D (2013). Characterization of gonadal transcriptomes from Nile tilapia (Oreochromis niloticus) reveals differentially expressed genes. PLoS One.

[21] Ye Z, Wang W, Zhang Y, Wang L, Cui Y, Li H (2020). Integrative analysis reveals pathways associated with sex reversal in Cynoglossus semilaevis. PeerJ.

[22] Nagasawa K, Thitiphuree T, Osada M (2019). Phenotypic stability of sex and expression of sex identification markers in the adult Yesso Scallop Mizuhopecten yessoensis throughout the reproductive cycle. Animals..

[23] Teaniniuraitemoana V, Huvet A, Levy P, Gaertner-Mazouni N, Gueguen Y, Le Moullac G (2015). Molecular signatures discriminating the male and the female sexual pathways in the pearl oyster Pinctada margaritifera. PLoS One.

[24] Galindo-Torres P, Garcia-Gasca A, Llera-Herrera R, Escobedo-Fregoso C, Abreu-Goodger C, Ibarra AM (2018). Sex determination and differentiation genes in a functional hermaphrodite scallop, Nodipecten subnodosus. Mar Genomics.

[25] Ashburner M, Ball CA, Blake JA, Botstein D, Butler H, Cherry JM, Davis AP, Dolinski K, Dwight SS, Eppig JT (2000). Gene ontology: tool for the unification of biology. The gene ontology consortium. Nat Genet.

[26] Takehana Y, Matsuda M, Myosho T, Suster ML, Kawakami K, Shin-I T, Kohara Y, Kuroki Y, Toyoda A, Fujiyama A, et al. Co-option of Sox3 as the male-determining factor on the Y chromosome in the fish Oryzias dancena. Nat Commun. 2014;5. 10.1038/ncomms5157 24948391

[27] Wu J, Xiong S, Jing J, Chen X, Wang W, Gui JF, Mei J (2015). Comparative Transcriptome analysis of differentially expressed genes and signaling pathways between XY and YY testis in yellow catfish. PLoS One.

[28] Wu JJ, Zhou YL, Wang ZW, Li GH, Jin FP, Cui LL, Gao HT, Li XP, Zhou L, Gui JF (2019). Comparative Transcriptome analysis reveals differentially expressed genes and signaling pathways between male and female red-tail catfish (Mystus wyckioides). Mar Biotechnol.

[29] Wong MC, Schwarzbauer JE (2012). Gonad morphogenesis and distal tip cell migration in the Caenorhabditis elegans hermaphrodite. Wiley Interdiscip Rev Dev Biol.

[30] Cabas I, Chaves-Pozo E, Alcazar AG, Meseguer J, Mulero V, Garcia-Ayala A (2011). Dietary intake of 17alpha-ethinylestradiol promotes leukocytes infiltration in the gonad of the hermaphrodite gilthead seabream. Mol Immunol.

[31] Menoud M, Van Wynsberge S, Le Moullac G, Levy P, Andrefouet S, Remoissenet G, Gaertner-Mazouni N (2016). Identifying robust proxies of gonad maturation for the Protandrous hermaphrodite Tridacna maxima (Roding, 1798, Bivalvia) from individual to population scale. J Shellfish Res.

[32] Shi Y, Liu W, He M (2018). Proteome and Transcriptome analysis of ovary, intersex gonads, and testis reveals potential key sex reversal/differentiation genes and mechanism in scallop Chlamys nobilis. Mar Biotechnol.

[33] Li Y, Zhang L, Sun Y, Ma X, Wang J, Li R, Zhang M, Wang S, Hu X, Bao Z (2016). Transcriptome sequencing and comparative analysis of ovary and testis identifies potential key sex-related genes and pathways in scallop Patinopecten yessoensis. Mar Biotechnol.

[34] Yu L, Xu D, Ye H, Yue H, Ooka S, Kondo H, Yazawa R, Takeuchi Y (2018). Gonadal Transcriptome analysis of Pacific abalone Haliotis discus discus: identification of genes involved in germ cell development. Mar Biotechnol.

[35] Tong Y, Zhang Y, Huang J, Xiao S, Zhang Y, Li J, Chen J, Yu Z (2015). Transcriptomics analysis of Crassostrea hongkongensis for the discovery of reproduction-related genes. PLoS One.

[36] Diz AP, Romero MR, Pérez-Figueroa A, Swanson WJ, Skibinski DOF (2018). RNA-seq data from mature male gonads of marine mussels Mytilus edulis and M. galloprovincialis. Data Brief.

[37] Zhang N, Xu F, Guo X (2014). Genomic Analysis of the Pacific Oyster (<em>*Crassostrea gigas*</em>) Reveals Possible Conservation of Vertebrate Sex Determination in a Mollusc. G3.

[38] Briones C, Nunez JJ, Perez M, Espinoza-Rojas D, Molina-Quiroz C, Guinez R (2018). De novo male gonad transcriptome draft for the marine mussel Perumytilus purpuratus with a focus on its reproductive-related proteins. J Genom.

[39] Yang D, Yin C, Chang YQ, Dou Y, Hao ZL, Ding J (2016). Transcriptome analysis of male and female mature gonads of Japanese scallop Patinopecten yessonsis. Genes Genom.

[40] Teaniniuraitemoana V, Huvet A, Levy P, Klopp C, Lhuillier E, Gaertner-Mazouni N, Gueguen Y, Le Moullac G (2014). Gonad transcriptome analysis of pearl oyster Pinctada margaritifera: identification of potential sex differentiation and sex determining genes. BMC Genomics.

[41] Grabherr MG, Haas BJ, Yassour M, Levin JZ, Thompson DA, Amit I, Adiconis X, Fan L, Raychowdhury R, Zeng QD (2011). Full-length transcriptome assembly from RNA-Seq data without a reference genome. Nat Biotechnol.

[42] Hedgecock D, Lin JZ, DeCola S, Haudenschild CD, Meyer E, Manahan DT, Bowen B (2007). Transcriptomic analysis of growth heterosis in larval Pacific oysters (Crassostrea gigas). P Natl Acad Sci USA.

[43] Clark MS, Thorne MAS, Vieira FA, Cardoso JCR, Power DM, Peck LS. Insights into shell deposition in the Antarctic bivalve Laternula elliptica: gene discovery in the mantle transcriptome using 454 pyrosequencing. BMC Genomics. 2010;11. 10.1186/1471-2164-11-362 PMC2896379 20529341

[44] Li CZ, Weng SP, Chen YG, Yu XQ, Lu L, Zhang HQ, He JG, Xu XP. Analysis of *Litopenaeus vannamei* Transcriptome Using the Next-Generation DNA Sequencing Technique. PLoS One. 2012;7(10):e47442. 10.1371/journal.pone.0047442 PMC3469548 23071809

[45] Hou R, Bao Z, Wang S, Su H, Li Y, Du H, Hu J, Wang S, Hu X (2011). Transcriptome sequencing and de novo analysis for Yesso scallop (Patinopecten yessoensis) using 454 GS FLX. PLoS One.

[46] Ho KK, Leung PT, Ip JC, Qiu JW, Leung KM (2014). De novo transcriptomic profile in the gonadal tissues of the intertidal whelk Reishia clavigera. Mar Pollut Bull.

[47] Li JY, Pan LQ, Miao JJ, Xu RY, Xu WJ (2016). De novo assembly and characterization of the ovarian transcriptome reveal mechanisms of the final maturation stage in Chinese scallop Chlamys farreri. Comp Biochem Phys D.

[48] Ogata H, Goto S, Sato K, Fujibuchi W, Bono H, Kanehisa M (1999). KEGG: Kyoto encyclopedia of genes and genomes. Nucleic Acids Res.

[49] Huo LJ, Fan HY, Liang CG, Yu LZ, Zhong ZS, Chen DY, Sun QY (2004). Regulation of ubiquitin-proteasome pathway on pig oocyte meiotic maturation and fertilization. Biol Reprod.

[50] Greenblatt EJ, Obniski R, Mical C, Spradling AC (2019). Prolonged ovarian storage of mature Drosophila oocytes dramatically increases meiotic spindle instability. Elife.

[51] Zheng Y, Quilliam LA (2003). Activation of the Ras superfamily of small GTPases. Workshop on exchange factors. EMBO Rep.

[52] Fuchs S, Behrends V, Bundy JG, Crisanti A, Nolan T (2014). Phenylalanine metabolism regulates reproduction and parasite melanization in the malaria mosquito. PLoS One.

[53] Wang Q, Cui Z, Guo H, Zhang N, Xu W, Yang Y, Chen S (2019). Functional analysis of the promoter of the dmrt1 gene in Chinese tongue sole, Cynoglossus semilaevis. J Oceanol Limnol.

[54] Webster KA, Schach U, Ordaz A, Steinfeld JS, Draper BW, Siegfried KR (2017). Dmrt1 is necessary for male sexual development in zebrafish. Dev Biol.

[55] Mawaribuchi S, Ito Y, Ito M: Independent evolution for sex determination and differentiation in the <em>DMRT</em> family in animals. Biol Open 2019, 8(8):bio041962. 10.1242/bio.041962 PMC6737965 31399444

[56] Naimi A, Martinez AS, Specq ML, Mrac A, Diss B, Mathieu M, Sourdaine P (2009). Identification and expression of a factor of the DM family in the oyster Crassostrea gigas. Comp Biochem Physiol A Mol Integr Physiol.

[57] Wang Q, Shi Y, He M (2018). Pf-Dmrt4, a potential factor in sexual development in the pearl oyster Pinctada fucata. J Oceanol Limnol.

[58] Tanaka SS, Nishinakamura R (2014). Regulation of male sex determination: genital ridge formation and Sry activation in mice. Cell Mol Life Sci.

[59] Sekido R, Lovell-Badge R (2008). Sex determination involves synergistic action of SRY and SF1 on a specific Sox9 enhancer. Nature.

[60] O'Brien EK, Degnan BM (2000). Expression of POU, sox, and Pax genes in the brain ganglia of the tropical abalone Haliotis asinina. Mar Biotechnol.

[61] Le Gouar M, Guillou A, Vervoort M (2004). Expression of a SoxB and a Wnt2/13 gene during the development of the mollusc Patella vulgata. Dev Genes Evol.

[62] Focareta L, Cole AG (2016). Analyses of sox-B and sox-E family genes in the cephalopod Sepia officinalis: revealing the conserved and the unusual. PLoS One.

[63] He Y, Bao Z, Guo H, Zhang Y, Zhang L, Wang S, Hu J, Hu X (2013). Molecular cloning and characterization of SoxB2 gene from Zhikong scallop Chlamys farreri. Chin J Oceanol Limnol.

[64] Li Y, Sosnik J, Brassard L, Reese M, Spiridonov NA, Bates TC, Johnson GR, Anguita J, Visconti PE, Salicioni AM (2011). Expression and localization of five members of the testis-specific serine kinase (Tssk) family in mouse and human sperm and testis. Mol Hum Reprod.

[65] Spiridonov NA, Wong L, Zerfas PM, Starost MF, Pack SD, Paweletz CP, Johnson GR (2005). Identification and characterization of SSTK, a serine/threonine protein kinase essential for male fertility. Mol Cell Biol.

[66] Shang P, Baarends WM, Hoogerbrugge J, Ooms MP, van Cappellen WA, de Jong AAW, Dohle GR, van Eenennaam H, Gossen JA, Grootegoed JA (2010). Functional transformation of the chromatoid body in mouse spermatids requires testis-specific serine/threonine kinases. J Cell Sci.

[67] Wang XL, Wei YH, Fu GL, Li HT, Saiyin H, Lin G, Wang ZG, Chen S, Yu L (2015). Tssk4 is essential for maintaining the structural integrity of sperm flagellum. Mol Hum Reprod.

[68] Sosnik J, Miranda PV, Spiridonov NA, Yoon S-Y, Fissore RA, Johnson GR, Visconti PE (2009). Tssk6 is required for Izumo relocalization and gamete fusion in the mouse. J Cell Sci.

[69] Zhou L, Liu Z, Dong Y, Sun X, Wu B, Yu T, Zheng Y, Yang A, Zhao Q, Zhao D (2019). Transcriptomics analysis revealing candidate genes and networks for sex differentiation of yesso scallop (Patinopecten yessoensis). BMC Genomics.

[70] Defaus S, Aviles M, Andreu D, Gutierrez-Gallego R (2016). Identification of bovine sperm surface proteins involved in carbohydrate-mediated fertilization interactions. Mol Cell Proteomics.

[71] Militz TA, Braley RD, Schoeman DS, Southgate PC (2019). Larval and early juvenile culture of two giant clam (Tridacninae) hybrids. Aquaculture.

[72] Modig C, Modesto T, Canario A, Cerda J, von Hofsten J, Olsson PE (2006). Molecular characterization and expression pattern of zona pellucida proteins in gilthead seabream (Sparus aurata). Biol Reprod.

[73] Zeng S, Gong Z (2002). Expressed sequence tag analysis of expression profiles of zebrafish testis and ovary. Gene.

[74] Yue HM, Cao H, Chen XH, Ye H, Li CJ, Du H (2014). Molecular characterization of the cDNAs of two zona pellucida genes in the Chinese sturgeon, Acipenser sinensis gray, 1835. J Appl Ichthyol.

[75] Liu X, Wang H, Gong Z (2006). Tandem-repeated Zebrafish zp3 genes possess oocyte-specific promoters and are insensitive to estrogen induction. Biol Reprod.

[76] Pisarska MD, Barlow G, Kuo F-T (2011). Minireview: roles of the forkhead transcription factor FOXL2 in granulosa cell biology and pathology. Endocrinology.

[77] Georges A, Auguste A, Bessiere L, Vanet A, Todeschini AL, Veitia RA (2014). FOXL2: a central transcription factor of the ovary. J Mol Endocrinol.

[78] Pannetier M, Fabre S, Batista F, Kocer A, Renault L, Jolivet G, Mandon-Pepin B, Cotinot C, Veitia R, Pailhoux E (2006). FOXL2 activates P450 aromatase gene transcription: towards a better characterization of the early steps of mammalian ovarian development. J Mol Endocrinol.

[79] Santerre C, Sourdaine P, Martinez AS (2012). Expression of a natural antisense transcript of cg-Foxl2 during the gonadic differentiation of the oyster Crassostrea gigas: first demonstration in the gonads of a lophotrochozoa species. Sex Dev.

[80] Liu XL, Li Y, Liu JG, Cui LB, Zhang ZF (2016). Gonadogenesis in scallop Chlamys farreri and Cf-foxl2 expression pattern during gonadal sex differentiation. Aquac Res.

[81] Mizuta H, Luo W, Ito Y, Mushirobira Y, Todo T, Hara A, Reading BJ, Sullivan CV, Hiramatsu N (2013). Ovarian expression and localization of a vitellogenin receptor with eight ligand binding repeats in the cutthroat trout (Oncorhynchus clarki). Comp Biochem Physiol Part B Biochem Mol Biol.

[82] Sarojini R, Nagabhushanam R, Fingerman M (1993). In-vivo evaluation of 5-Hydroxytryptamine stimulation of the testes in the fiddler-crab, Uca-Pugilator - a presumed action on the neuroendocrine system. Comp Biochem Phys C.

[83] Laufer H, Biggers WJ, Ahl JSB (1998). Stimulation of ovarian maturation in the crayfish Procambarus clarkiiby methyl Farnesoate. Gen Comp Endocrinol.

[84] Kulkarni GK, Fingerman M (1992). Quantitative analysis by reverse phase high performance liquid chromatography of 5-Hydroxytryptamine in the central nervous system of the red swamp crayfish, Procambarus clarkii. Biol Bull.

[85] Fingerman M (1997). Roles of neurotransmitters in regulating reproductive hormone release and gonadal maturation in decapod crustaceans. Invert Reprod Dev.

[86] Maran C, Tassone E, Masola V, Onisto M (2009). The story of SPATA2 (spermatogenesis-associated protein 2): from Sertoli cells to pancreatic Beta-cells. Curr Genomics.

[87] Liu SF, He S, Liu BW, Zhao Y, Wang Z (2004). Cloning and characterization of testis-specific spermatogenesis associated gene homologous to human SPATA4 in rat. Biol Pharm Bull.

[88] Xie M-C, Ai C, Jin X-M, Liu S-F, Tao S-X, Li Z-D, Wang Z (2007). Cloning and characterization of chicken SPATA4 gene and analysis of its specific expression. Mol Cell Biochem.

[89] Yuan S, Stratton CJ, Bao J, Zheng H, Bhetwal BP, Yanagimachi R (2015). Yan W: <em>Spata6</em> is required for normal assembly of the sperm connecting piece and tight head–tail conjunction. Proc Natl Acad Sci.

[90] Huo S, Du W, Shi P, Si Y, Zhao S (2015). The role of spermatogenesis-associated protein 6 in testicular germ cell tumors. Int J Clin Exp Pathol.

[91] Li WZ, Godzik A (2006). Cd-hit: a fast program for clustering and comparing large sets of protein or nucleotide sequences. Bioinformatics.

[92] Waterhouse RM, Seppey M, Simao FA, Manni M, Ioannidis P, Klioutchnikov G, Kriventseva EV, Zdobnov EM (2018). BUSCO applications from quality assessments to gene prediction and Phylogenomics. Mol Biol Evol.

[93] Langmead B. Aligning short sequencing reads with Bowtie. Curr Protocols Bioinform. 2010;32:11.7.1–14. 10.1002/0471250953.bi1107s32 PMC3010897 21154709

[94] Mortazavi A, Williams BA, McCue K, Schaeffer L, Wold B (2008). Mapping and quantifying mammalian transcriptomes by RNA-Seq. Nat Methods.

[95] Anders S, McCarthy DJ, Chen YS, Okoniewski M, Smyth GK, Huber W, Robinson MD (2013). Count-based differential expression analysis of RNA sequencing data using R and bioconductor. Nat Protoc.

[96] Beissbarth T, Speed TP (2004). GOstat: find statistically overrepresented gene ontologies within a group of genes. Bioinformatics.

[97] Livak KJ, Schmittgen TD (2001). Analysis of relative gene expression data using real-time quantitative PCR and the 2−ΔΔCT method. Methods.

