# Supplementary material for: Comparative transcriptome analysis of three gonadal development stages reveals potential genes involved in gametogenesis of the fluted giant clam (Tridacna squamosa)
Source: BMC Genomics. 2020 Dec 7;21:872. doi: 10.1186/s12864-020-07276-5 (PMC7720611; doi:10.1186/s12864-020-07276-5)
Supplement: Supplementary file 1 — Additional file 1: Table S1. Sequences of primers used in this study [file 12864_2020_7276_MOESM1_ESM.doc]

**Table S1**

Sequences of primers used in this study

| **Gene ID** | Sequence (5'-3') |
| --- | --- |
| DMRT-F | CTGATTTTACGTGGTTGTGG |
| DMRT-R | AATGCAGATTTAAACGAAC |
| SPAPA17-F | CAGAAAATCCCTATGATCCT |
| SPAPA17-R | GCCTCCTCCAGGGATGTATAT |
| SOX8-F  SOX8-R | GTTTTACGCTGATGTCAGAA  GGGAAAAAGTGTACTGCTG |
| TSSK1-F | AACATCACTTTCTTTTCCAG |
| TSSK1-R  SP17-F  SP17-R  ZP-F  ZP-R  FOXL2-F  FOXL2-R  5HTR-F  5HTR-R  VR-F  VR-R  ATRX-F  ATRX-R  EF1 | ACATTTTGTGGGAGCGCCG  GCTGCACAGAAAAATATCGCAA  ATATCCCCAACATAGTCAC  CCCTTATTTGAAATCTCTG  TTGAATCTTGGGTACATCCA  TGGACATAATTCGGCCACCGG  GGGTACCGAGGGGTAGTCA  CAAACTATCTTATCCTATCGC  CAGTAGCGGTCCACCGAGAT  CTTGAGCCGGGCCTGTTCTT  ATGTAATGGTATTGTGGACTGT  TCAGAACTGAATTTCTCAGA  ACATTTTGTTGTTGTGAGGG  TCTTCGGTCAATCTTCTCTT  GGAGATCAAGAGAGGAAAT |

“F” indicates forward primer and “R” indicates reverse primer.
