# Supplementary material for: Comparative transcriptome analysis of three gonadal development stages reveals potential genes involved in gametogenesis of the fluted giant clam (Tridacna squamosa)
Source: BMC Genomics. 2020 Dec 7;21:872. doi: 10.1186/s12864-020-07276-5 (PMC7720611; doi:10.1186/s12864-020-07276-5)
Supplement: Supplementary file 2 — Additional file 2: Table S2. Correlation analysis between different gonadal development groups. [file 12864_2020_7276_MOESM2_ESM.docx]

**Table S2**

Correlation analysis between different gonadal development groups.

| Sample | Resting 1 | Resting 2 | Resting 3 | Male 1 | Male 2 | Male 3 | Hermaphrodite 1 | Hermaphrodite 2 | Hermaphrodite 3 |
| --- | --- | --- | --- | --- | --- | --- | --- | --- | --- |
| Resting 1 | 1 |  |  |  |  |  |  |  |  |
| Resting 2 | 0.9407 | 1 |  |  |  |  |  |  |  |
| Resting 3 | 0.8959 | 0.9382 | 1 |  |  |  |  |  |  |
| Male 1 | 0.2655 | 0.2583 | 0.2329 | 1 |  |  |  |  |  |
| Male 2 | 0.1820 | 0.1759 | 0.1649 | 0.9498 | 1 |  |  |  |  |
| Male 3 | 0.3980 | 0.3981 | 0.3643 | 0.8294 | 0.8123 | 1 |  |  |  |
| Hermaphrodite 1 | 0.5535 | 0.5217 | 0.5037 | 0.7135 | 0.6564 | 0.5669 | 1 |  |  |
| Hermaphrodite 2 | 0.784 | 0.7321 | 0.6719 | 0.4651 | 0.3712 | 0.5155 | 0.8142 | 1 |  |
| Hermaphrodite 3 | 0.5651 | 0.5254 | 0.4816 | 0.6003 | 0.5086 | 0.6910 | 0.8591 | 0.8031 | 1 |

Values shown represent r^2^ value. The condition (r^2^>0.80) was used to eliminate differential individuals.
