# Supplementary figures and images for: Comparative transcriptome analysis of three gonadal development stages reveals potential genes involved in gametogenesis of the fluted giant clam (Tridacna squamosa)
Source: BMC Genomics. 2020 Dec 7;21:872. doi: 10.1186/s12864-020-07276-5 (PMC7720611; doi:10.1186/s12864-020-07276-5)

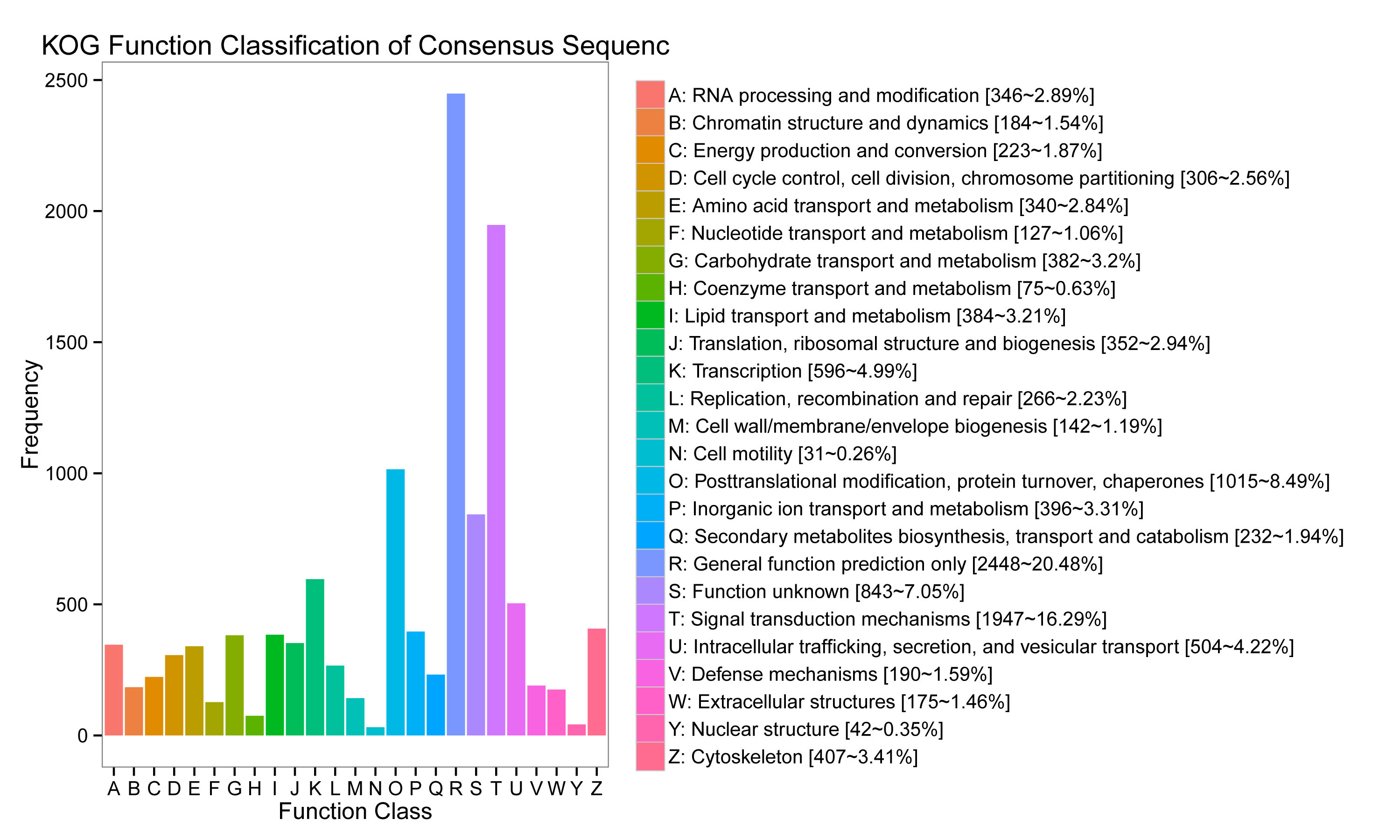

Supplement: Supplementary file 6 — Additional file 6: Figure S1. Clusters of orthologous group (COG) function classifications of T. squamosa. The x-axis shows 25 categories, while the y-axis shows the number of DEGs corresponding to each category. [file 12864_2020_7276_MOESM6_ESM.jpg]

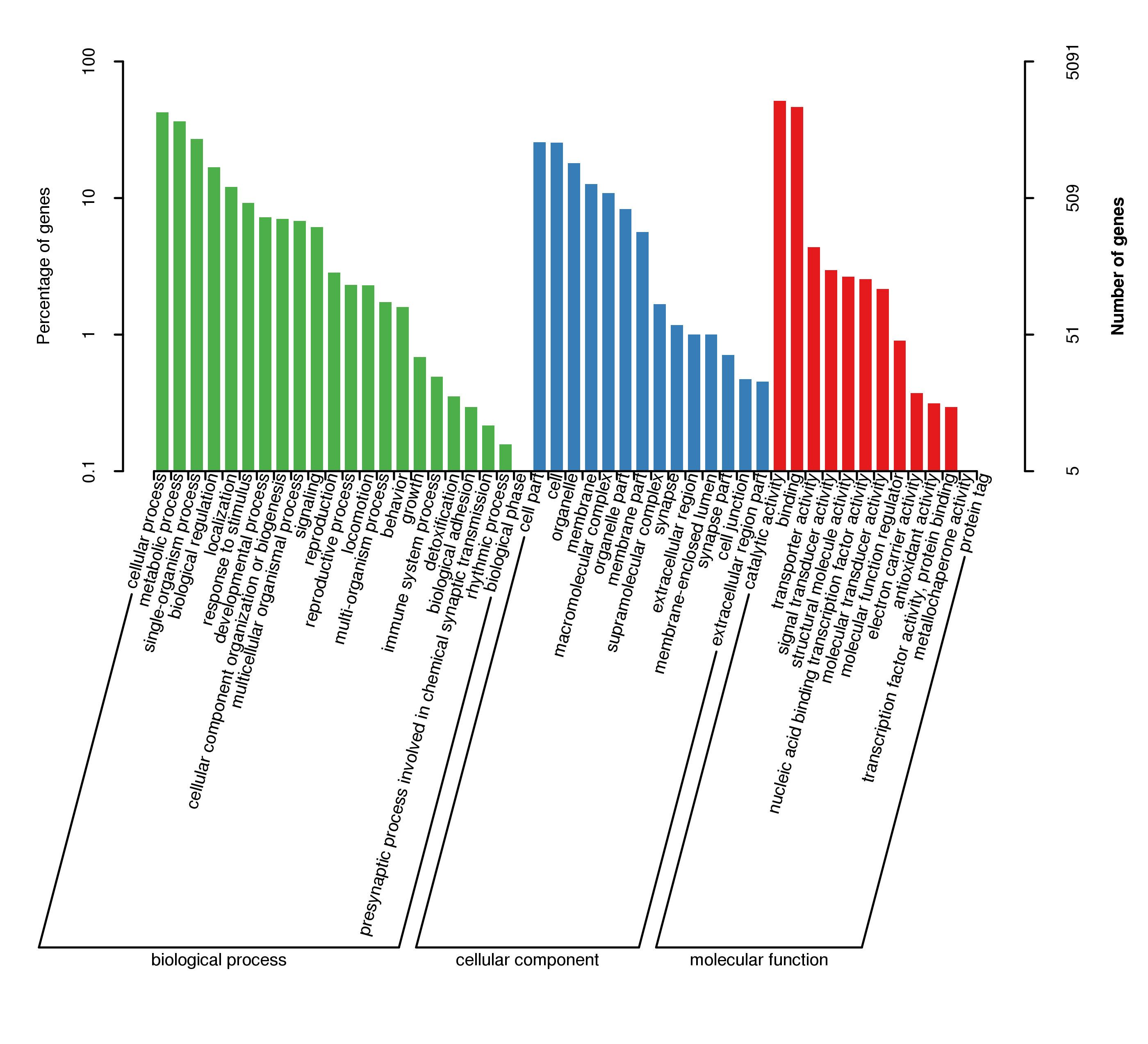

Supplement: Supplementary file 7 — Additional file 7: Figure S2. GO distributions of T. squamosa transcriptomes. [file 12864_2020_7276_MOESM7_ESM.jpg]
